# Analysis of exome sequence in 604 trios for recessive genotypes in schizophrenia

**DOI:** 10.1038/tp.2015.99

**Published:** 2015-07-21

**Authors:** E Rees, G Kirov, J T Walters, A L Richards, D Howrigan, D H Kavanagh, A J Pocklington, M Fromer, D M Ruderfer, L Georgieva, N Carrera, P Gormley, P Palta, H Williams, S Dwyer, J S Johnson, P Roussos, D D Barker, E Banks, V Milanova, S A Rose, K Chambert, M Mahajan, E M Scolnick, J L Moran, M T Tsuang, S J Glatt, W J Chen, H-G Hwu, The Taiwanese Trios Exome Sequencing Consortium, Stephen V Faraone, Cheri A Roe, Sharon D Chandler, Chih-Min Liu, Chen-Chung Liu, Ling-Ling Yeh, Wen-Chen Ouyang, Hung-Yu Chan, Chun-Ying Chen, B M Neale, A Palotie, P Sklar, S M Purcell, S A McCarroll, P Holmans, M J Owen, M C O'Donovan

**Affiliations:** 1 Medical Research Council Centre for Neuropsychiatric Genetics and Genomics, Institute of Psychological Medicine and Clinical Neurosciences, Cardiff University, Cardiff, UK; 2 Analytic and Translational Genetics Unit, Department of Medicine, Massachusetts General Hospital and Harvard Medical School, Boston, MA, USA; 3 Division of Psychiatric Genomics, Department of Psychiatry, Institute for Genomics and Multiscale Biology, Icahn School of Medicine at Mount Sinai, New York, NY, USA; 4 Stanley Center for Psychiatric Research, Broad Institute of MIT and Harvard, Cambridge, MA, USA; 5 Program in Medical and Population Genetics, Broad Institute of MIT and Harvard, Cambridge, MA, USA; 6 Institute for Molecular Medicine Finland, University of Helsinki, Helsinki, Finland; 7 GOSgene,ICH Genetics and Genomic Medicine Program, Institute of Child Health, Faculty of Population Health Sciences, University College London, London, UK; 8 Molecular Genetics and Genomics, National Heart and Lung Institute, Imperial College London, London, UK; 9 Department of Psychiatry, Medical University, Sofia, Bulgaria; 10 Center for Behavioral Genomics and Institute for Genomic Medicine, University of California, San Diego, La Jolla, CA, USA; 11 Department of Psychiatry, University of California, San Diego, La Jolla, CA, USA; 12 Harvard Institute of Psychiatric Epidemiology and Genetics, Boston, MA, USA; 13 Psychiatric Genetic Epidemiology and Neurobiology Laboratory (PsychGENe Lab); Department of Psychiatry and Behavioral Sciences, SUNY Upstate Medical University, Syracuse, NY, USA; 14 Institute of Epidemiology and Preventive Medicine, College of Public Health, National Taiwan University, Taipei, Taiwan; 15 Department of Psychiatry, National Taiwan University Hospital, College of Medicine, National Taiwan University, Taipei, Taiwan; 16 Department of Genetics, Harvard Medical School, Boston, MA, USA; 17 Medical Research Council Centre for Neuropsychiatric Genetics and Genomics, Neuroscience and Mental Health Research Institute, Institute of Psychological Medicine and Clinical Neurosciences, Cardiff University, Cardiff, UK

## Abstract

Genetic associations involving both rare and common alleles have been reported for schizophrenia but there have been no systematic scans for rare recessive genotypes using fully phased trio data. Here, we use exome sequencing in 604 schizophrenia proband–parent trios to investigate the role of recessive (homozygous or compound heterozygous) nonsynonymous genotypes in the disorder. The burden of recessive genotypes was not significantly increased in probands at either a genome-wide level or in any individual gene after adjustment for multiple testing. At a system level, probands had an excess of nonsynonymous compound heterozygous genotypes (minor allele frequency, MAF ⩽1%) in voltage-gated sodium channels (VGSCs; eight in probands and none in parents, *P*=1.5 × 10^−^^4^). Previous findings of multiple *de novo* loss-of-function mutations in this gene family, particularly *SCN2A*, in autism and intellectual disability provide biological and genetic plausibility for this finding. Pointing further to the involvement of VGSCs in schizophrenia, we found that these genes were enriched for nonsynonymous mutations (MAF ⩽0.1%) in cases genotyped using an exome array, (5585 schizophrenia cases and 8103 controls), and that in the trios data, synaptic proteins interacting with VGSCs were also enriched for both compound heterozygosity (*P*=0.018) and *de novo* mutations (*P*=0.04). However, we were unable to replicate the specific association with compound heterozygosity at VGSCs in an independent sample of Taiwanese schizophrenia trios (*N*=614). We conclude that recessive genotypes do not appear to make a substantial contribution to schizophrenia at a genome-wide level. Although multiple lines of evidence, including several from this study, suggest that rare mutations in VGSCs contribute to the disorder, in the absence of replication of the original findings regarding compound heterozygosity, this conclusion requires evaluation in a larger sample of trios.

## Introduction

High heritability points to a major role for an inherited genetic contribution to the aetiology of schizophrenia. A large number of rare^1, 2, 3^ and common^4, 5^ schizophrenia risk alleles are known to exist. Risk variants are widely but not randomly dispersed across the genome, rather they are relatively enriched in genes encoding proteins involved in synaptic function, particularly the activity-regulated cytoskeleton-associated protein and *N*-methyl-D-aspartate receptor complexes, fragile X mental retardation protein targets and voltage-gated calcium ion channels.^1, 2, 6^ Risk variants are also enriched in genes that are expressed in immune tissues,^7^ even after excluding those that are also expressed in the brain.^7^ Given the heterogeneity of the disorder and the large number of genes involved, it is very likely that additional biological processes are involved in the disorder.

Most studies have implicitly assumed additive or dominant models of inheritance. Given the genetic complexity of schizophrenia, it is also reasonable to postulate that some alleles may act in a recessive manner, a hypothesis that has support in autism spectrum disorder (ASD).^8^ Recessive inheritance occurs either via homozygosity (two copies of the same allele) or compound heterozygosity (two different mutations, one on each parental chromosome). Homozygosity is particularly likely in the offspring of consanguineous parents. Some studies suggest that schizophrenia is also enriched in the offspring of consanguineous parents, circumstantially supporting the hypothesis of homozygous recessive inheritance.^9, 10, 11^ More direct evidence comes from observations of an excess of genome-wide autozygosity (homozygous DNA segments that are identical by descent) in some^12, 13^ but not all^14^ studies of schizophrenia.

In outbred populations, compound heterozygosity rather than homozygosity is the more likely explanation for recessive inheritance, particularly in the context of substantial genetic heterogeneity.^15^ However, this form of inheritance is difficult to study in the unrelated case and control samples that have become the focus of most genomics work in schizophrenia. Although a compound heterozygous status can sometimes be distinguished probabilistically from the alternative genotype status (where the two mutant sites occur on the same parental chromosome) without parental genotype information,^8^ this process is sensitive to allele frequency. Under the most extreme low frequency scenario where pairs of mutant alleles occur only once, they cannot be phased. As damaging alleles are disproportionately represented among rare variants,^16, 17^ this may be an important limitation for studying compound heterozygosity in case–control samples. In contrast, family-based studies allow compound heterozygosity to be directly observed; if both parents carry only a single mutant allele at a gene, then offspring with both copies must be compound heterozygotes at that gene.

Two studies of recessive inheritance in schizophrenia, both using the same case–control exome-sequencing data set, did not provide any significant evidence for these genotypes contributing to schizophrenia risk.^18, 19^ Here, we conduct the first investigation of recessive inheritance in schizophrenia using exome-sequencing data from a parent–offspring trio sample (*N*=604), adopting the principles of gene-set analyses that have been informative in rare variant studies of the disorder to date.^1, 2, 6^ Novel gene sets showing evidence for associations in the Bulgarian trios were further examined for (a) rare variant enrichment using independent unpublished exome array data (5585 schizophrenia cases and 8103 controls), (b) *de novo* mutation enrichment in published schizophrenia, ASD, intellectual disability (ID) and control data sets and (c) rare, recessive genotypes in phase 1 of an independent schizophrenia parent–offspring trio sample (*N*=614).

## Materials and methods

### Samples

The schizophrenia parent–proband trio sample has been described previously.^1, 6^ More detail is provided in the Supplementary Material (section 2). Briefly, probands with schizophrenia (*N*=534) or schizoaffective disorder (*N*=89) were recruited from psychiatric hospitals in Bulgaria. All the probands had attended and graduated from mainstream schools, which at the time in Bulgaria excluded people known to have a significant ID or developmental delay. The SCAN instrument^20^ was used to perform a semi-structured interview for psychosis and mood symptoms and consensus diagnoses were made by two clinicians according to DSM-IV criteria. The 623 probands, of which 306 are male, comprise 597 trios (proband and parents), 12 quads (two affected children) and one multi-generational family (affected daughter of an affected mother).

### Sequencing and variant calling

The details for sequencing and variant calling in the Bulgarian trios have been described previously^1^ and in the Supplementary Material (section 3). Briefly, paired-end whole-exome sequencing on Illumina HiSeq sequencers was performed across three centres: The Broad Institute, the Icahn School of Medicine at Mount Sinai and the Wellcome Trust Sanger Institute. All unmapped sequence reads were aligned to the human reference genome (hg19) and variants were called at the Broad Institute using the BWA/Picard/GATK pipeline. A description of our method used to call compound heterozygous genotypes can be found in the Supplementary Material (section 3, Supplementary Figures S1 and S2).

### Variant annotation

Using ANNOVAR software,^21^ all the variants were annotated for their predicted functional consequence (for example, synonymous/nonsynonymous) according to RefSeq transcripts, for whether they overlapped a segmental duplication, their predicted effect on protein function (Sift,^22^ PolyPhen 2 (HumDiv)^23^ and MutationTaster^24^) and for their frequency in the 1000 genomes project (all combined populations, 2012 release).^25^ In addition, variants were annotated for their frequency among all individuals in the exome variant server (combined European and African American populations, ESP6500 release^26^) and for their frequency among the Bulgarian parental chromosomes.

### Variant filtering and quality control

Each variant site was required to pass the following filters in all members of a trio where at least one individual was a carrier: sequencing depth ⩾10; genotyping quality score ⩾30; alternative allele balance ⩽0.8 and ⩾0.2 for heterozygous calls, ⩾0.9 for homozygous calls of the non-reference allele and ⩽0.1 for homozygous calls of the reference allele. Thresholds were selected empirically to ensure there was no significant distortion of exome-wide transmission from the null expectation (50:50) for variants with a minor allele frequency (MAF) of ⩽1% (global transmission disequilibrium test, *P*=0.07, transmission:non-transmission ratio=0.99). All the variants within segmental duplications were excluded as these are known to be enriched for sequencing artefacts. We did not include indels as their calling is still less reliable than for point mutations. Given that our study was designed to test genes for rare pathogenic recessive genotypes, we excluded *TTN* and *MUC16* from our analyses as these genes had large numbers of rare (MAF ⩽1%) recessive genotypes of our sample (*n*=291 and 121, respectively).

Five trios were outliers with respect to the exome-wide rate of transmitted alleles and were excluded from all the analyses (Supplementary Material, section 4 and Supplementary Figure S4). We also excluded an additional trio, which was shown to have poor quality calls in our previous study.^1^ After removing low quality trios and related probands, 604 probands from 604 families were retained for analysis: from the initial 623 probands, six were excluded for quality and 13 were excluded for being related (one proband from the 12 quads and one proband from the multi-generational family).

### Global enrichment analysis

To test for an excess of recessive genotypes in the probands, we followed the method of Lim *et al.*^8^ Here, a normalized enrichment ratio was calculated as (*N* genotypes in probands/*N* genotypes in parents) × (*N* parents/*N* probands). The statistical significance of a global enrichment in probands was evaluated with a one-sided test by randomizing proband/parent status for 10 000 permutations. Our primary analyses were performed using an MAF threshold of ⩽1% as rarer alleles are enriched for more damaging mutations.^16^ To allow comparisons with a recent autism study,^8^ we additionally present the results for analyses at MAF ⩽5%. In the autosomal analysis, variants were excluded if they exceeded the given MAF threshold in either the 1000 genomes project, EVS or among Bulgarian parental chromosomes. For the analysis of X chromosomes, variants were excluded if they exceeded the given MAF threshold among mothers in the Bulgarian trio sample. In all the analyses, we included only nonsynonymous point mutations.

### Gene-set analysis

We undertook a ‘candidate' gene-set analysis based upon a composite of sets significantly enriched for rare mutations in recent schizophrenia exome-sequencing studies^1, 2^ (Supplementary Table S1). Seeking novel insights into the disorder, we also undertook a non-hypothesis pathway analysis based upon Gene Ontology (GO) annotations (Supplementary Table S2). Gene to GO annotations were derived from NCBI gene2go, using Homo Sapiens annotations only. AmiGO ontology (http://www.geneontology.org/GO.downloads.ontology.shtml) was used to calculate each GO terms parent term. Child–parents relationships between terms were defined using ‘is_a' and ‘part_of' (but not ‘regulates'). The parent terms of each GO term assigned to a gene in gene2go were also assigned to that gene. GO terms were tested if they contained three or more genes, and >5 recessive genotypes were observed in the Bulgarian trio sample. Gene-set analyses were performed separately for compound heterozygous, homozygous and all recessive genotypes. Based upon the results of the global burden test, our primary analysis focussed on nonsynonymous compound heterozygous genotypes at an MAF ⩽1%, although we present the complete set of results in the Supplementary Material.

To test for an excess in probands over parents of recessive genotypes, a similar analysis approach was used to that described in Kirov *et al.*^6^ Briefly, the change in deviance was compared with a one-sided analysis of variance test between the following two logistic regression models:

#### Model 1

Logit (pr(proband))~*N* recessive genotypes in gene set+*N* recessive genotypes outside gene set+sequencing site/batch

#### Model 2

Logit (pr(proband))~*N* recessive genotypes outside gene set+sequencing site/batch

The inclusion of *N* recessive genotypes outside the gene set corrects for differences in exome-wide burden of recessive genotypes between probands and parents. Samples were processed in six batches at the Icahn School of Medicine at Mount Sinai and at the Wellcome Trust Sanger Institute, and in eight batches at The Broad Institute. All Bulgarian family members were processed in the same batch. We included a covariate for sequencing site (The Broad Institute, The Icahn School of Medicine at Mount Sinai or The Wellcome Trust Sanger Institute) and sequencing batch to control for potential technical variation across sites and batches. In the candidate gene-set analysis, *P*-values were corrected for multiple testing by randomly permuting proband/parent status, repeating the logistic regression analysis for all the pathways tested and comparing the test *P*-value with the most significant pathway *P*-value generated in the permuted data. In all, 10 000 permutations were used to generate corrected *P-*values. The same permutation approach was used to generate pathway-specific *P*-values when the number of recessive genotypes hitting the pathway was small (making asymptotic distributions unreliable). As running permutations for the large number of sets in the GO pathway analysis was computationally impractical, we note in the results whether any gene set survives Bonferroni correction for multiple testing.

### Exome array data

The exome chip is designed to genotype rare coding variants previously observed in sequencing data sets (http://genome.sph.umich.edu/wiki/Exome_Chip_Design). Our exome array sample contains 5585 schizophrenia cases (ascertainment described in Rees *et al.*^3^) and 8103 controls taken from the UK Blood Service and the 1958 British Birth Cohorts.^27, 28, 29^ These control samples have not been screened for psychiatric illness. The full details of this sample and analysis are given in a parallel manuscript (Richards *et al*, in preparation). Briefly, SKAT-O^30^ was used to examine genes and/or gene sets, which were significantly associated with schizophrenia in the analysis of recessive inheritance in the Bulgarian trios. The exome chip analysis was limited to nonsynonymous single-nucleotide polymorphisms with an MAF ⩽0.1%. A lower frequency threshold is used here as we are not testing a recessive model (see above comments about phasing), and variants with an MAF ⩽0.1% showed the strongest evidence for enrichment in a recent schizophrenia exome-sequencing study.^2^

### *De novo* gene-set enrichment analysis

*De novo* mutations from controls and from individuals with schizophrenia, ASD and ID were derived from multiple publications as summarized in Fromer *et al.*^1^ The method used to test for *de novo* gene-set enrichment has been described previously^1^ and allows for the probability of *N* *de novo* mutations occurring in a gene set of length (*S*) adjusted for sequencing coverage. Random placement of the observed number of *de novo* mutations was used to generate the expected number of mutations in the gene set under the null. The following equation, as described in Fromer *et al.,*^1^ was used to calculate gene-set enrichment *P*-values:


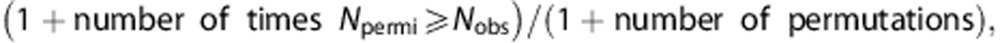


where *N*_permi_ is the number of randomly generated mutations in a given gene set and *N*_obs_ is the number of observed mutations in a given gene set.

### Inbreeding coefficients

The inbreeding coefficient (F statistic) for each sample was generated with PLINK^31^ using previously published single-nucleotide polymorphism genotyping data.^6, 7^

#### Taiwanese parent–proband sample

Replication of significant findings in the analysis of the Bulgarian trios were sought in phase 1 of a Taiwanese schizophrenia parent–offspring sample (*N*=614 trios). Full details on the ascertainment and sequencing of the Taiwanese parent–proband replication sample can be found in the Supplementary Material (section 2). Briefly, the Taiwanese sample was sequenced at the Broad Institute using paired-end whole-exome sequencing performed on Illumina HiSeq sequencers. Similar to the Bulgarian cohort, reads were aligned with human reference genome (hg19) and variants called using the BWA/Picard/GATK pipeline. Recessive genotypes were called and analysed with the same methods used for the Bulgarian trios. Variants in the Taiwanese sample were excluded if they had a MAF greater than a given threshold in either 1000 genomes project (all populations), EVS (all populations) or among Taiwanese parental chromosomes. Filtering variants by MAFs derived from Taiwanese parental chromosomes will account for allele frequency differences that exist between Taiwanese and European populations.

## Results

### Inbreeding

Probands were not inbred relative to their parents, indicating that the latter is a suitable control for the former with respect to homozygosity (proband median F=0.00373, parent median F=0.00375, Mann–Whitney *U* *P*=0.72).

### Exome-wide burden of recessive genotypes

In autosomes, there was no significant excess of recessive genotypes in probands after correcting for multiple testing (homozygous, compound heterozygous and all recessive genotypes, two variant types and two MAF thresholds), although there was a nominally significant excess in probands of rare (MAF ⩽1%) nonsynonymous compound heterozygosity (Table 1). Our sample had 99% power to detect a significant (*P*=0.05) twofold excess of loss-of-function (LOF) recessive genotypes (MAF ⩽5%) in probands versus parents as reported in ASD.^8^ For rare (MAF ⩽1%) nonsynonymous compound heterozygous mutations, our sample has 80% power to detect a mean rate difference of 0.30 between probands and parents at a significance that corrects for multiple testing (*P*=4.2 × 10^−3^). An exploratory analysis restricted to nonsynonymous variants most likely to disrupt protein function (stop gain/splice annotations and missense variants predicted to be damaging by three algorithms) similarly revealed no evidence for global enrichment for recessive mutations (Supplementary Table S5, section 5). The X chromosome mutational burden did not significantly differ for hemizygous alleles when male probands were compared with fathers or for homozygous genotypes when female probands were compared with mothers (Supplementary Table S6, section 6). These results did not change when significance was tested by permuting proband/parent status within families rather than across all families (data not shown).

### Enrichment analyses

Following the results of our burden analysis, our primary enrichment analysis tested rare (MAF ⩽1%) nonsynonymous compound heterozygous genotypes, with the remaining genotype classes and MAF thresholds presented as secondary analyses. The primary analysis identified no genes with a significant excess in probands of nonsynonymous compound heterozygosity after adjusting for the total number of genes studied (Supplementary Table S3). This was also true for the secondary analyses of different recessive genotypes or MAF thresholds (Supplementary Table S3). Our top gene for all rare (MAF ⩽1%), nonsynonymous recessive genotypes is *BLM* (five in four probands and none in parents). *BLM* encodes the protein RecQL_3_ that repairs aberrant DNA replication.^32^ Autosomal recessive genotypes in *BLM* cause Bloom syndrome (#210900), an extremely rare disorder characterized by chromosomal instability, malignancies, short stature, dermatological conditions and reduced immunoglobulins IgM and IgA.^32^ Of the five recessive genotypes we observe in our Bulgarian cohort, only one involved LOF alleles. The Bulgarian proband who was homozygous for two different alleles in *BLM*, one LOF and the other missense, was diagnosed with catatonic schizophrenia, but also had vitiligo and epilepsy. The number of probands with *BLM* recessive genotypes observed here is too small to draw conclusions about their role in schizophrenia, but we provide phenotypic details on these probands in Supplementary Table S7.

We found no significant enrichments for rare (MAF ⩽1%) nonsynonymous compound heterozygous genotypes in the composite candidate gene set or its constituent parts (Supplementary Table S1). Several of the secondary analyses (that is, recessive genotypes other than compound heterozygous with MAF ⩽1%) showed nominally significant enrichments, such as nonsynonymous compound heterozygous genotypes (MAF ⩽5%) in postsynaptic density genes and nonsynonymous homozygous genotypes (MAF ⩽5% and MAF ⩽1%) in genes disrupted by *de novo* LOF mutations in studies of ASD, although none of these survived correction for multiple testing (Supplementary Table S1). Given the small number of LOF recessive genotypes and nonsynonymous recessive genotypes predicted to be damaging observed in the Bulgarian cohort, we do not present the enrichment results for these genotypes, but note that no set was significant after correction for multiple testing.

The results of the larger gene ontology set analysis are presented in Supplementary Table S2. The top three gene sets in our primary analysis of nonsynonymous compound heterozygous genotypes (MAF ⩽1%) are non-independent sodium channel sets (Supplementary Table S2). The evidence for association for each of these gene sets derives from the same set of eight compound heterozygous genotypes in probands versus none in parents (*P*=1.77 × 10^−5^). We focused all subsequent analyses on the voltage-gated sodium channel complex (VGSC) set as it contains the minimum set of genes (*N*=14) common to all the three sodium channel sets. Through permutation testing, we determined an association *P*-value of 1.5 × 10^−4^ for this gene set, a result that does not survive Bonferroni correction for the number of GO sets tested for rare (MAF ⩽1%) compound heterozygous genotypes. We note that Bonferroni correction is likely to be over-conservative given that certain GO sets are not completely independent. The variants involved in these VGSC compound heterozygous genotypes are presented in Supplementary Table S4.

Overall, nonsynonymous variants (MAF ⩽1%) were not over-transmitted from heterozygous parents at VGSCs (*P*=0.65, Supplementary Table S8, section 8), indicating that the above finding was specific to compound heterozygosity rather than a consequence of general over-transmission under additive or dominant models.

We next sought independent evidence for association between VGSC genes and schizophrenia in exome chip data from 5585 schizophrenia cases and 8103 controls. VGSC genes were enriched for rare (MAF ⩽0.1%) nonsynonymous variants (*P*=0.01), with 2 of the 14 genes showing nominally significant enrichment—*SCN3A* (*P*=0.001) and *SCN4A* (*P*=0.01) (Supplementary Table S9, section 9). We also tested VGSCs for enrichment of *de novo* mutations previously reported in schizophrenia, ASD, ID and control phenotypes. Strong enrichments were found for LOF *de novo* mutations in ASD and ID, largely driven by *de novo* mutations in *SCN2A*, a known gene for various developmental disorders,^33, 34, 35^ although no significant enrichments were found in schizophrenia or control populations (Supplementary Table S10, section 10). A full breakdown of which VGSC genes were disrupted by *de novo* mutations is provided in Supplementary Table S9. In addition, we found synaptic proteins that interact with VGSCs, which were identified in the synaptic interactome available from the SynSysNet database (further details in Supplementary Material, section 11), to be enriched for rare (MAF ⩽1%) nonsynonymous compound heterozygous genotypes (*P*=0.018, odds ratio=2, Supplementary Table S11). This enrichment in probands remained significant after correcting for the number of rare (MAF ⩽1%) nonsynonymous compound heterozygous genotypes observed in synaptic proteins not found to interact with VGSCs (*P*=0.032, Supplementary Material, section 11). Synaptic proteins interacting with VGSCs were also enriched for schizophrenia *de novo* LOF mutations (*P*=0.04, Supplementary Table S13).

We attempted to replicate our original association of compound heterozygosity in VGSCs in phase 1 of an independent Taiwanese schizophrenia parent–proband sample (*N*=614). Here, rare (MAF ⩽1%) compound heterozygous genotypes were not enriched in the probands when compared with parents (Table 2). None of the Taiwanese parents with a VGSC compound heterozygous genotype had received a diagnosis of schizophrenia. Association between compound heterozygosity in VGSCs and schizophrenia retained nominal significance in a combined analysis of the Bulgarian and Taiwanese trios (Table 2).

## Discussion

In a sample of over 600 schizophrenia trios, we did not observe an increased burden of recessive genotypes in affected probands at an exome-wide level or in any specific gene. Significant findings from previous studies of rare mutation in schizophrenia have resulted from analyses of ultra-rare (MAF ⩽0.1%) alleles under a dominant model.^2^ Our analyses of recessive inheritance were conducted with a higher predefined MAF threshold (⩽1% and ⩽5%). However, our conclusions would not change if our analyses were conducted on alleles with an MAF of ⩽0.1% (data not shown). A recent autism study reported a significantly higher rate (twofold) of autosomal recessive LOF genotypes (MAF ⩽5%) in cases compared with controls.^8^ We estimate that our schizophrenia sample has 99% power at a significance threshold of *P*=0.05 to detect a twofold enrichment of autosomal recessive LOF genotypes (MAF ⩽5%). However, similar to a previous study,^19^ we do not find a significant excess of LOF recessive genotypes in schizophrenia. We observed a nominally significant excess in probands versus parents of rare (MAF ⩽1%) nonsynonymous compound heterozygous genotypes, with our sample having 80% power to detect a corrected significant mean rate difference of 0.30 between probands and parents. Therefore, given our sample size, our study does not have the power to ‘exclude' a modest contribution of this class of allele. Based upon findings from similar studies of rare variants in schizophrenia,^2^ it is unsurprising that we did not implicate any individual gene, but we note that our most significant gene for all recessive genotypes (MAF ⩽1%) is *BLM*, which causes a known disorder (Bloom syndrome #210900). Our failure to identify an overall excess of rare recessive genotypes at the levels of the exome or of single genes is similar to findings from a much larger exome-sequencing study (which did not look at recessive inheritance),^2^ analyses of recessive genotypes in the same schizophrenia exome-sequencing case–control sample^18, 19^ and our own analysis of *de novo* exonic mutations in the present Bulgarian sample.^1^

We next sought evidence for an excess of recessive genotypes at the level of gene sets, an approach that has been successful in many studies of rare mutations, including earlier exome-sequencing studies of schizophrenia.^1, 2^ Here, the rationale is that biologically relevant pathways should have greater enrichment for mutations than the exome average, thus improving signal-to-noise ratio, while at the same time, gene sets may contain enough genes to permit multiple observations of events that are too rare at the gene level to be detected in current samples.

Our analysis of candidate gene sets did not find any significant associations, which could be due to a lack of power, or reflect the fact that recessive genotypes in these genes are not enriched in schizophrenia. In our gene ontology set analysis, where we sought to identify novel gene sets associated with schizophrenia, our most significant result was for nonsynonymous compound heterozygous genotypes (MAF ⩽1%) in VGSCs. We note that half of the variants involved in these VGSCs were singleton observations and therefore could only have been phased with trio data. The VGSC set comprises 14 genes: 10 structurally related alpha subunits that form transmembrane ion channels responsible for the generation and propagation of electrical signals in excitable cells, such as neurons, and four beta-subunits which associate with the channels to modulate their kinetics.^33, 36^

Mutations in VGSCs are a known cause of neurological disorders such as Dravet syndrome (MIM #607208),^37^ and multiple *de novo* mutations in *SCN1A*, *SCN2A* and *SCN8A* have also been observed in patients with epileptic encephalopathies,^38^ ASD^34, 35^ and ID.^39, 40^ None of the probands in the current Bulgarian trio sample with a compound heterozygous genotype in a VGSC suffered from epilepsy. Also, Bulgarian proband carriers of VGSC compound heterozygous genotypes are not different from proband non-carriers with regards to age of onset, diagnosis (schizoaffective disorder/schizophrenia) or gender (Supplementary Table S14).

We found two independent lines of evidence suggesting a role for VGSCs in schizophrenia. First, we found evidence of association in a case–control analysis of variants (MAF ⩽0.1%) called from exome chip arrays, indicating that risk alleles in VGSCs might not always act recessively. Second, we found significant associations for rare (MAF ⩽1%) nonsynonymous compound heterozygous and *de novo* LOF mutations in VGSC-interacting genes and schizophrenia, suggesting their disruption could also increase schizophrenia risk through impacting sodium channel function.

In an attempt to support our original association between compound heterozygosity in VGSCs and schizophrenia, we analysed phase 1 of an independent schizophrenia trio sample from Taiwan. Given that the enrichment in the Bulgarian probands of compound heterozygous genotypes in VGSCs primarily involved singleton alleles, it was important that our replication attempt was also conducted using parent–proband trio data to allow accurate phasing of rare alleles. The Taiwanese sample did not lend support to the association between compound heterozygosity in VGSC and schizophrenia. Although this replication attempt could have been confounded by population or unknown clinical phenotype differences between the Bulgarian and Taiwanese samples, the results clearly suggest a need for further data before drawing definitive conclusions on the association between VGSCs and schizophrenia.

A major strength of the current study is the ability to phase all alleles in the probands using their parental genotypes, thus providing the first accurate assessment of ultra-rare compound heterozygosity in schizophrenia. Also, by using the parents as a control population, our analysis should be unaffected by population stratification.

In conclusion, rare, recessive genotypes do not appear to substantially contribute to schizophrenia genetic liability. We provide further evidence that LOF recessive genotypes (MAF ⩽5%) are not significantly associated with schizophrenia.^19^ Several independent lines of evidence indicate that rare variants in voltage-gated sodium channel genes increase the risk of developing schizophrenia, although further work is needed before this gene set can be confidently implicated in the disorder.

## Figures and Tables

**Table 1:** Number of autosomal compound heterozygous (compHet), homozygous and all recessive genotypes (compHet+homozygous) observed among probands and parents in the Bulgarian sample

*Variant (MAF)*	*Genotype*	*Probands* N *mut. (rate)*	*Parents* N *mut. (rate)*	P_*uncorrected*_	P_*corrected*_
NS (⩽1%)	CompHet	1485 (2.46)	2790 (2.31)	0.030	0.43
	Homozygous	488 (0.81)	1049 (0.87)	0.76	1
	All recessive	1973 (3.27)	3839 (3.18)	0.25	1
LOF (⩽1%)	CompHet	4 (0.0066)	6 (0.0050)	0.43	1
	Homozygous	17 (0.028)	28 (0.023)	0.23	1
	All recessive	21 (0.035)	34 (0.028)	0.25	1
					
NS (⩽5%)	CompHet	5869 (9.72)	11 740 (9.72)	0.50	1
	Homozygous	3046 (5.04)	6322 (5.23)	0.85	1
	All recessive	8915 (14.76)	18 062 (14.95)	0.78	1
LOF (⩽5%)	CompHet	7 (0.012)	8 (0.0066)	0.09	1
	Homozygous	52 (0.086)	84 (0.070)	0.12	1
	All recessive	59 (0.098)	92 (0.076)	0.07	0.84

Abbreviations: LOF, loss of function; MAF, minor allele frequency; mut., mutant; NS, nonsynonymous.

**Table 2:** Rare (MAF ⩽1%) nonsynonymous compound heterozygous genotypes in voltage-gated sodium channels

*Genotype*	*Bulgarian trios*			*Taiwanese trios*			*Combined sample* P-*value*
	*Probands* N=*604*	*Parents* N=*1208*	P-*value*	*Probands* N=*614*	*Parents* N=*1228*	P-*value*	
*N* VGSC CompHets (rate)	8 (0.013)	0 (0)	1.5 × 10^−4^	3 (0.0049)	9 (0.0073)	0.73	0.024

Abbreviations: compHets, compound heterozygous; MAF, minor allele frequency; VGSC, voltage-gated sodium channel.
