# Supplementary material for: Analysis of exome sequence in 604 trios for recessive genotypes in schizophrenia
Source: Transl Psychiatry. 2015 Jul 21;5(7):e607–. doi: 10.1038/tp.2015.99 (PMC5068730; doi:10.1038/tp.2015.99)
Supplement: Supplementary Information [file tp201599x1.doc]

**Supplementary Material: Analysis of exome sequence in 604 trios for recessive genotypes in schizophrenia**

**Table of contents**

1. Extended supplementary tables.
2. Sample description.
3. Sequencing and variant calling.
4. Identifying trios with low quality sequence data
5. Analysis of autosomal nonsynonymous variants predicted to be damaging
6. X chromosome burden of recessive genotypes.
7. Phenotypic details of probands with *BLM* recessive genotypes
8. Transmission disequilibrium test in voltage-gated sodium channel genes.
9. Summary of voltage-gated sodium channels in different genetic datasets.
10. Enrichment of *de novo* mutations in voltage-gated sodium channels
11. Enrichments among voltage-gated sodium channel-interacting genes.
12. Phenotypic description of probands with a voltage-gated sodium channel compound heterozygous genotype.
13. **Extended Supplementary tables**

**Table S1.** Candidate gene set analysis. Analysis of gene sets previously associated with schizophrenia in exome sequencing studies (Fromer *et al* 2014^1^, Purcell *et al* 2014^2^). Results are presented for nonsynonymous compound heterozygous, homozygous and all recessive genotypes under two minor allele frequency (MAF) thresholds (≤1% and ≤5%).

**Table S2**. Gene ontology set analysis. Nonsynonymous compound heterozygous, homozygous and all recessive genotypes under two MAF thresholds (≤1% and ≤5%) are tested in a non-hypothesis analysis of all Gene Ontology (GO) sets.

**Table S3.** Single gene enrichment analysis. Each RefSeq gene was tested with a 1-sided Fisher’s Exact test for being enriched among probands for nonsynonymous compound heterozygous, homozygous and all recessive genotypes under two MAF thresholds (≤1% and ≤5%). Results are shown for genes enriched among probands with at least nominal levels of significance (*P* < 0.05).

**Table S4.** Compound heterozygous genotypes (MAF ≤1%) in voltage-gated sodium channels discovered in the Bulgarian trios.

1. **Sample description**

Bulgarian trios

The Bulgarian schizophrenia proband-parent sample has been described previously^1, 3^. The sample consisted of 534 probands with schizophrenia, 89 probands with schizoaffective disorder and their parents recruited from several hospitals in Bulgaria. All probands were interviewed for psychotic and mood symptoms using the SCAN instrument and consensus diagnoses were made by two clinicians according to DSM-IV criteria. When a different diagnosis was made between clinicians, the patient was further interviewed by a research interview trained clinician and excluded if a consensus diagnosis could still not be reached. The 623 probands, of which 306 are male, comprise 597 trios (proband and parents), 12 quads (two affected children) and one multi-generational family (affected daughter of an affected mother). Bulgarian families were prioritised for exome-sequencing if they had a negative family history for psychiatric illness. Of the 1208 parents in the families that survived quality control and were analysed for recessive genotypes, a psychiatric diagnosis was recorded for only 7.

Taiwanese trios

**Clinical ascertainment approach**

The clinical ascertainment of the probands and the trio family was comprised of three stages: The first stage was obtaining approvals from all of the IRBs of the hospitals participating in this study for (1) recruiting the proband and trio family, and the data and sample collections, and for (2) the approval of the content of the informed consent documents for both the proband and the parents. The major content of the informed consent form was composed of the following elements: goal of this study, inclusion/exclusion criteria of the study subjects, methods of this study (diagnostic interview, blood sample collections), management of any remaining sample (e.g. DNA samples), potential adverse effects of study activities and its management, the expected results, the requested activities of the recruited participants of this study, confidentiality, compensation and insurance of the participants for any adverse effect encountered in this study, rights of the recruited participants, withdrawal and termination for participating this study, and de-linking the basic identifiable personal data from the sample.

The second stage was the recruitment of the probands and the parents over a 5-year period starting in June, 2009 and concluding in March, 2014. Recruitment including clinical screening and obtaining informed consent.

(1)    Clinical screening, using a clinical screening sheet, was employed to (a) exclude those potential subjects with an ancestor of aboriginal origin; (b) include those potential subjects fulfilling the DSM-IV criteria of schizophrenia for proband recruitment based on clinical observation and interview by the attending psychiatrist providing the psychiatric services.

(2)    After identifying the potential proband, the parents were informed about the details of this study, and initial oral consent was obtained. The proband and the parents were then given the IRB-approved consent forms and the details of this study were explained. We then obtained the signed informed consent documents, and the proband joined this study.

The third stage was the collection of clinical data and diagnostic assessment.

(1)    A DIGS interview of the proband was performed by well-trained research assistants, with backgrounds in nursing, psychology or social work.  Following the interview, a review of the clinical chart records in the hospital or in the clinical service settings was made prior to completing the clinical summary by the research assistants.

(2)    The blood samples were drawn for DNA analysis.

(3)    Initial research diagnostic assessment was performed based on integrated clinical information of DIGS interview data and summary note of clinical course, symptom manifestations and social functioning derived from the records of the medical charts. Two board-certified psychiatrists independently completed the initial research diagnostic assessments.  If both research diagnostic assessments reached a consensus diagnosis of schizophrenia, the research diagnosis was finalized. If there was a discrepancy in the diagnostic assessment, then the case was subject to the last step of research diagnosis assessment.

(4)    The last research diagnostic assessment was done by the senior research psychiatrist (Professor Hai-Gwo Hwu) based on the information of clinical screening sheet, the DIGS interview data, and the clinical summary note. If necessary, the research psychiatrist would call up the field attending psychiatrists for clarification of the clinical information crucial for the diagnostic assessment. Twenty-seven subjects were excluded from this study due to the diagnostic deviation from schizophrenia (DSM-IV). After this exclusion, a total of 3008 trio families were retained in this sample.

**Sites of ascertainment**

The headquarters of this Taiwan Trio family study was located in the Department of Psychiatry, National Taiwan University Hospital and College of Medicine, National Taiwan University, Taipei, Taiwan. The island country of Taiwan is situated in the Pacific Ocean about 160 km from the southern coast of the Chinese mainland. The population size of Taiwan is 23,271,643 (September, 2014), and it has the surface area of 36,188 km^2^. All mental hospitals, community care centres and the primary care clinics in Taiwan were enrolled as potential sites for proband recruitment. The recruitment catchment areas included metropolitan Taipei (population 7,030,620) , Northern Taiwan (population 3,588,318), Middle Taiwan (population 4,520,155), Southern Taiwan (population 3,387.035), KaoPing area (population 3,728,269) and the Eastern Taiwan (population 1,017,246). The number of clinical settings participating in the recruitment activity was 76, 28, 45, 31, 36, and 24 respectively in these 6 recruitment areas. The successfully recruited probands of the trio families was 704, 558, 569, 576, 497 and 104, respectively.  The recruitment ratio of probands/10,000 population are 1.0, 1.5, 1.26, 1.70, 1.33, and 1.02, respectively for these 6 recruitment catchment areas.

**Parental phenotype**

The affected status of the father and mother was designated as yes or no only by the family history method. No personal interview with parents was performed. None of the Taiwanese parents in families selected for exome sequencing in the current study had received a diagnosis of schizophrenia.

**DNA source and extraction**

Rutger’s University Cell and DNA Repository

**Duration (years) of sample collection**

6 years

**IRB / Ethics approval / Consent** **verification**

Most of the 813 IRB approvals are in Chinese. They can be provided upon request. The UCSD IRB Authorization was #081600 and approval and consents can be sent.

**Funding of collection**

NIMH R01 MH085521 and R01 MH085560

1. **Sequencing and variant calling**

Sequencing and variant calling in the Bulgarian trio sample has been described previously^1^. Each Bulgarian trio was randomly assigned to The Broad Institute, Mount Sinai School of Medicine or the Wellcome Trust Sanger Institute where whole-exome sequencing was performed. The samples had their exomes captured using either Agilent hybrid capture or Nimblegen array-based capture and were subjected to paired-end sequencing on Illumina HiSeq sequencers. All members of the same trio were sequenced at the same site using the same exome capture. Therefore, cases (probands) and controls (parents) were matched for exome capture. Of the Bulgarian trio samples, 92% had a mean sequencing coverage of at least 70x. A median of 93% of targeted exome bases were covered by 10 or more reads. All unmapped sequence reads were processed and called for variants at the Broad Institute using the BWA/Picard/GATK pipeline. Further details regarding sequencing coverage and number of variants called can be found at Fromer *et al* 2014^1^. The Bulgarian trio sequence data is available from dbGaP (phs000687.v1.p1).

**Calling homozygous and compound heterozygous genotypes**

Samples homozygous for an alternative allele are identified during the GATK variant calling pipeline. To increase confidence that homozygous calls are correctly distinguished from heterozygous calls, individuals homozygous for the reference and non-reference allele had to pass an allele balance (number of alternative reads/total number of reads) filter of less than 0.1 and greater than 0.9, respectively.

The identification of compound heterozygous genotypes requires the phase of alleles to be known. The phase of alleles in the probands can be directly observed since the parental genotypes are also known. Therefore, probands were defined as having a compound heterozygous genotype in a particular gene if they had inherited at least two different variants within that gene on each parental chromosome (Figure S1). The false positive rate of compound heterozygous genotype calls in probands is likely to be extremely low given all variants contributing to these genotypes had to be observed in both parent and proband.


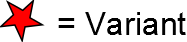

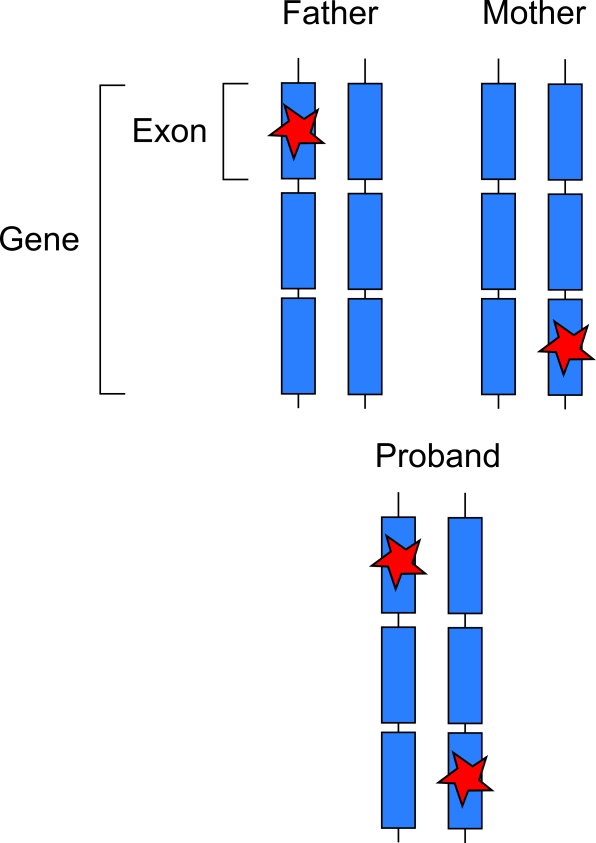


**Figure S1.** Schematic of a proband with a compound heterozygous genotype. The proband has inherited a variant from each parent at different sites within a gene.

Variants in the parents cannot be phased in the same way alleles are phased in probands unless the genotypes of the grandparents are also known. Therefore, parents were called as having a compound heterozygous genotype if they were observed to transmit one allele and not transmit a different allele, indicating these alleles reside on different chromosomes (Figure S2). It is possible for two variants to occupy the same parental chromosome with only one of them being transmitted to the child if they have been separated by recombination. However, given the small genomic distance separating any two variants within the same gene, the chances of a recombination occurring between them will be very small.

All the variants in probands analysed for compound heterozygosity were required to have their parent of origin known. For example, a site found to be heterozygous in all members of trio cannot be analysed for compound heterozygosity as the parent which transmitted the allele cannot be distinguished from the parent which did not transmit the allele.


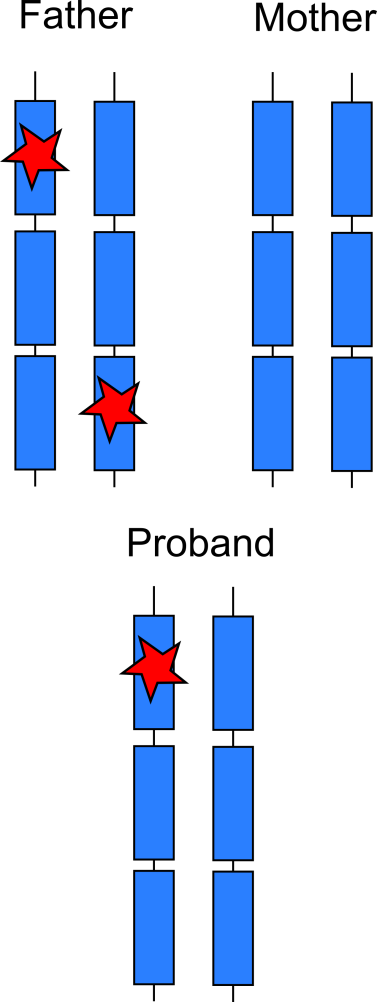


**Figure S2**. Schematic of a father with a compound heterozygous genotype. The proband has inherited one of the two heterozygous variants from the father. Therefore, we assume the variants in the father reside on different chromosomes.

We were able to test our method of calling compound heterozygous genotypes in parents using exome-sequencing data from a multi-generation family (Figure S3). Here, compound heterozygous genotypes were first predicted in the mother using alleles she did or did not transmit to her daughter using the method described above. These predicted compound heterozygous genotypes were then compared to those directly observed using alleles transmitted from the grandparents. For variant sites that passed QC in all 5 family members, all 17 compound heterozygous genotypes directly observed in the mother using alleles transmitted from the grandparents were predicted using transmitted and non-transmitted alleles to the daughter.


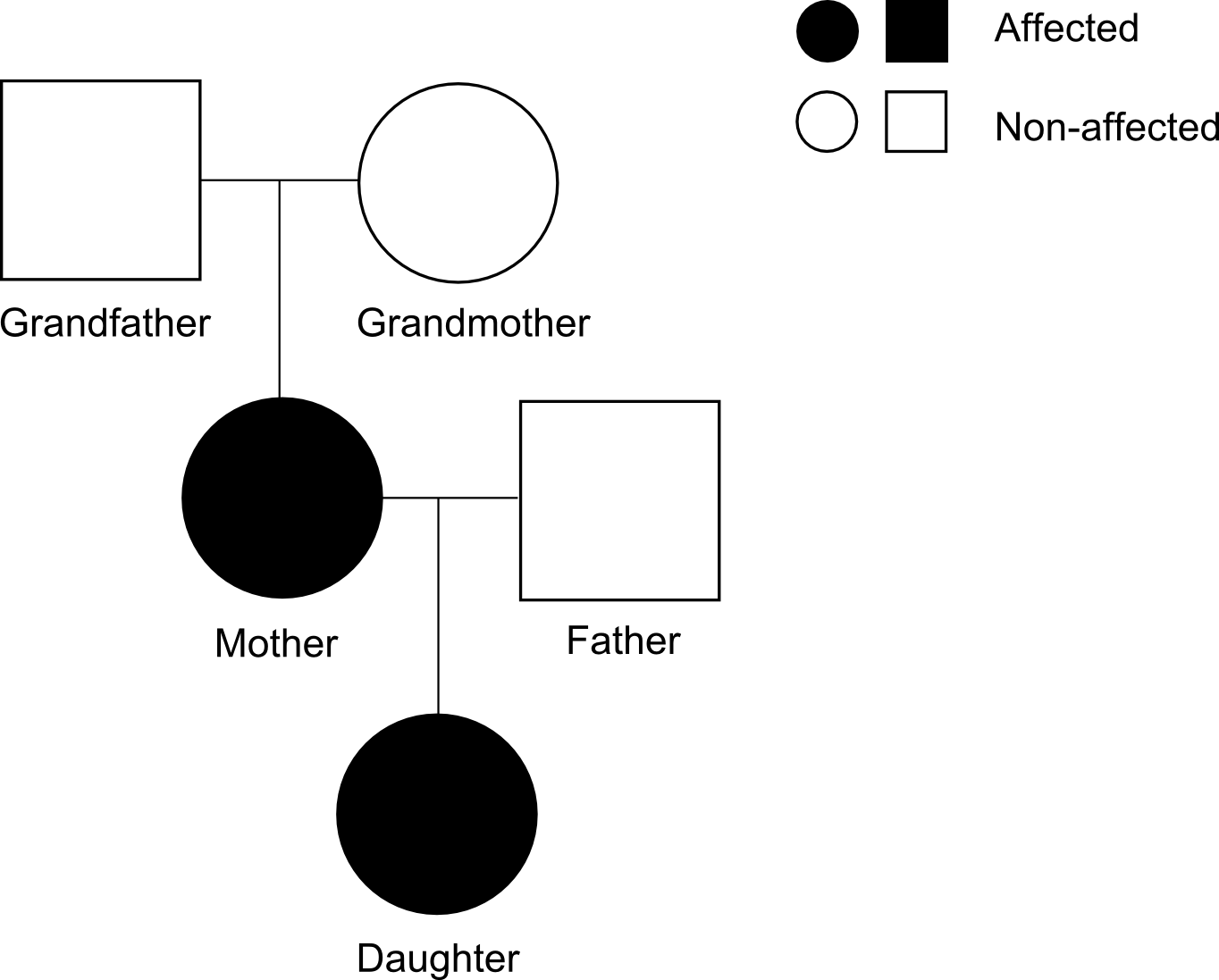


**Figure S3**. Multi-generation family used to test method of calling compound heterozygous genotypes in parents.

1. **Identifying trios with low quality sequence data**

To identify trios with low quality sequence data and/or non-familial relationships, we tested the per-trio transmission/non-transmission rate of rare (MAF≤1%) nonsynonymous variants from heterozygous parents. Here, low quality sequence data and/or non-familial relationships should result in an excess non-transmission. This method identified five trios as being outliers with respect to excess non-transmission (Figure S4). These trios were excluded from all analyses.


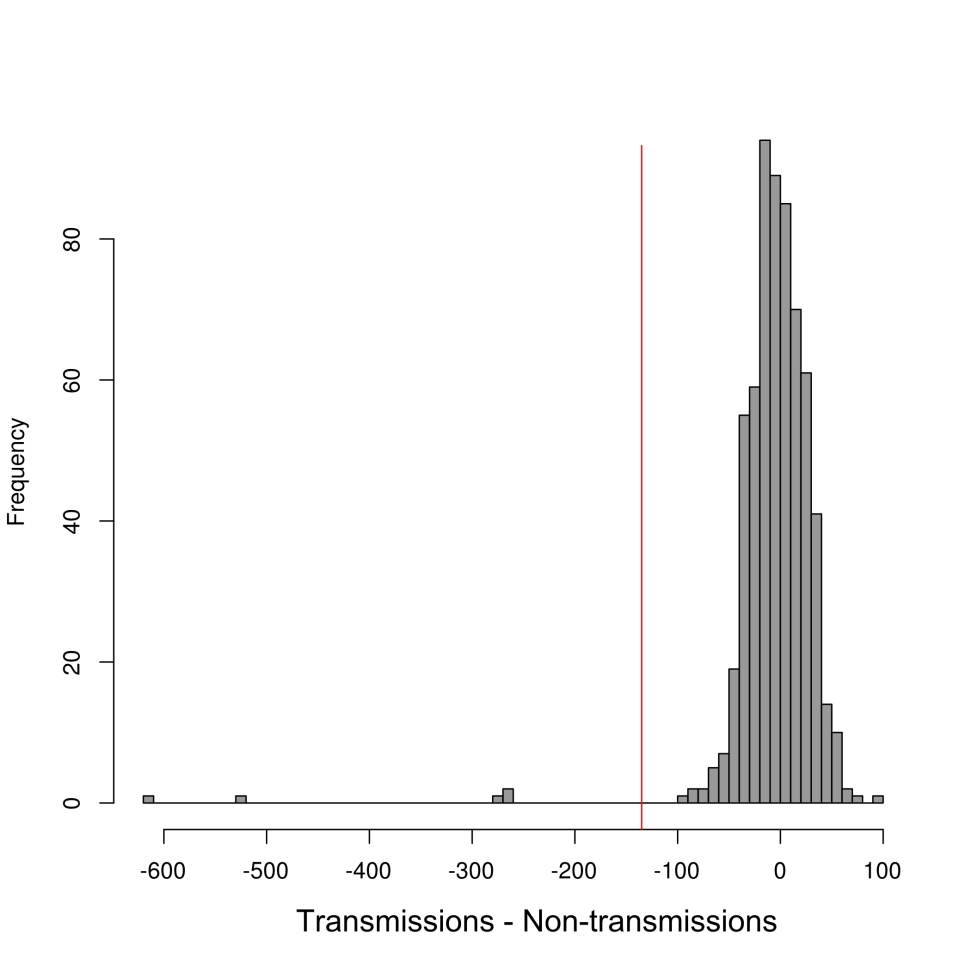


**Figure S4.** Assessment of trio sequencing quality. X-axis shows the number of transmissions minus non-transmissions for each trio. A substantial excess of non-transmitted alleles should be observed in trios with low quality sequencing data. Trios were excluded from our analysis if their excess non-transmission was greater than three standard deviations (indicated by red line).

1. **Analysis of autosomal nonsynonymous variants predicted to be damaging**

We tested for enrichment of recessive genotypes in probands compared with parents for autosomal variants predicted to have a damaging effect on protein function. These variants included all loss-of-function point mutations (stop-gain and splice annotations) and missense point mutations predicted to be damaging by three different algorithms (Sift^4^, PolyPhen2 (HumDiv)^5^ and MutationTaster^6^). We did not find any evidence that the rate of damaging recessive genotypes differed between probands and parents (Table S5).

| **Variant (MAF)** | **Genotype** | **Probands**  **N mut. (Rate)** | **Parents**  **N mut. (Rate)** | ***P*_uncorrected_** | **P_corrected_** |
| --- | --- | --- | --- | --- | --- |
| NS damaging (≤1%) | CompHet | 81 (0.13) | 155 (0.13) | 0.40 | 1 |
|  | Homozygous | 97 (0.16) | 188 (0.16) | 0.44 | 1 |
|  | All Recessive | 178 (0.29) | 343 (0.28) | 0.38 | 1 |
| NS damaging (≤5%) | CompHet | 232 (0.38) | 471 (0.39) | 0.59 | 1 |
|  | Homozygous | 420 (0.70) | 835 (0.69) | 0.47 | 1 |
|  | All Recessive | 652 (1.08) | 1 306 (1.08) | 0.53 | 1 |

**Table S5.** Number of damaging nonsynonymous (NS) autosomal compound heterozygous (compHet), homozygous and all recessive genotypes (compHet + homozygous) observed among probands and parents in the Bulgarian sample. Recessive genotypes were classed as damaging if they only involved loss-of-function alleles or missense alleles predicted to be damaging by Sift^4^, PolyPhen2 (HumDiv)^5^ and MutationTaster^6^.

1. **X chromosome burden of recessive genotypes**

To test for an overrepresentation of X chromosome recessive genotypes in the schizophrenia probands, we compared the number of hemizygous alleles observed in male probands with fathers and the number of homozygous genotypes observed in female probands with mothers (Table S6). No significance difference between probands and parents was observed for either of these tests for alleles with a MAF ≤5% or ≤1%.

| Test | Variant (MAF) | Probands  N mut. (rate) | Parents  N mut. (rate) | P_uncorrected_ |
| --- | --- | --- | --- | --- |
| Male probands (296)  vs  Male parents (604) | NS (≤5%) | 1,647 (5.56) | 3,259 (5.40) | 0.21 |
|  | NS (≤1%) | 916 (3.09) | 1,784 (2.95) | 0.17 |
|  | LOF (≤5%) | 46 (0.16) | 98 (0.16) | 0.64 |
|  | LOF (≤1%) | 14 (0.047) | 25 (0.041) | 0.40 |
| Female probands (308)  vs  Female parents (604) | NS (≤5%) | 40 (0.13) | 95 (0.16) | 0.72 |
|  | NS (≤1%) | 12 (0.039) | 17 (0.028) | 0.31 |
|  | LOF (≤5%) | 2 (0.0065) | 1 (0.0017) | 0.26 |
|  | LOF (≤1%) | 0 (0) | 1 (0.0017) | 1 |

**Table S6.** X chromosome burden of hemizygous alleles in male samples and homozygous genotypes in female samples.

1. **Phenotypic details of probands with *BLM* recessive genotypes**

| **Proband** | **Genotype** | **Diagnosis** | **Gender** | **AOO** | **Hallucinations** | **Delusions** | **Negative symptoms** | **Quality of remissions** | **Other symptoms** |
| --- | --- | --- | --- | --- | --- | --- | --- | --- | --- |
| 1 | Compound heterozygous  Chr15:91304476, A/G. miss  Chr15:91346808, G/C, miss | SZ, paranoid subtype. | Female | 15 | Voices, olfactory | Grandeur, fantastic, systematised, bizarre. | Present, severe | Chronic course |  |
| 2 | Compound heterozygous  Chr15:91293043, C/T, miss  Chr15:91306241, G/A, miss | SZ, paranoid subtype. | Male | 23 | Voices, olfactory. | Reference, grandeur, misidentification, fantastic, being controlled, systematised. Delusions were completely incongruent. |  | Partial remission |  |
| 3 | Compound heterozygous  Chr15:91308570, C/T, miss  Chr15:91347463, T/A, miss | SZ, disorganised subtype. | Female | 16 | Voices | Both congruent and incongruent | Present, severe | Chronic course |  |
| 4 | Homozygous  Chr15:91308570, C/T, miss  Homozygous  Chr15:91304245, C/T, LOF | SZ, unspecified | Female | 23 | Voices | Congruent. Variable insight Catatonic symptoms |  | First episode | Vitiligo and epilepsy. |

**Table S7**. Phenotypic description of probands carrying a recessive mutation in *BLM*. Miss = missense, LOF = loss-of-function, AOO = age-of-onset.

1. **Transmission disequilibrium test in voltage-gated sodium channel genes**

The results from transmission disequilibrium tests for variants in voltage-gated sodium channels (VGSCs) are shown in Table S8. We did not observe a significant deviation from the null for nonsynonymous variants with a MAF ≤1%, indicating a low false positive rate of variants called within these genes (a high false positive rate would result in an excess non-transmission) and that the excess compound heterozygosity in VGSCs is not driven by general over-transmission.

| **Variant (MAF)** | **Transmissions** | **Non-transmissions** | **P_uncorrected_** |
| --- | --- | --- | --- |
| NS (≤1%) | 295 | 284 | 0.65 |
| NS (Singletons) | 86 | 100 | 0.30 |
| LOF (≤1%) | 4 | 1 | 0.18 |
| LOF (Singletons) | 4 | 1 | 0.18 |

**Table S8.** Transmission disequilibrium test (TDT) of nonsynonymous (NS) and loss-of-function (LOF) variants in voltage-gated sodium channel genes.

1. **Summary of mutations in voltage-gated sodium channels in different genetic datasets**

A breakdown of the number of compound heterozygous, *de novo* and loss-of-function heterozygous mutations in VGSCs that were found in the Bulgarian trios, as well as compound heterozygous genotypes in the Taiwanese trios and gene-wide *P* values from a SCAT-O analysis of rare (MAF≤0.1%) nonsynonymous alleles 5,585 independent schizophrenia cases and 8,103 independent controls, is provided in Table S9.

| Gene | In SynSysNet | NS Compound hets  ≤1% frequency BG trios | | NS Compound hets  ≤1% frequency TW trios | | *De novo* mutation  (Missense/LOF counts) | | | | SZ Bulgarian trio  Heterozygous TDT | | SZ Exome chip |
| --- | --- | --- | --- | --- | --- | --- | --- | --- | --- | --- | --- | --- |
|  |  | Probands  (N = 604) | Parents  (N = 1,208) | Probands  (N = 614) | Parents  (N = 1,228) | SZ | ASD | ID | ASD sibling +  controls | LOF T | LOF NT | *P*-value |
| SCN10A |  | 1 |  |  | 3 |  |  |  |  |  |  | 1.0000 |
| SCN11A |  | 2 |  | 1 | 1 |  |  |  |  | 1 | 1 | 0.3015 |
| SCN1A | Presynaptic |  |  |  |  |  | 1/0 |  |  |  |  | 0.1284 |
| SCN1B | Presynaptic |  |  |  |  |  |  |  |  |  |  | NA |
| SCN2A | Presynaptic |  |  |  |  | 0/1 | 1/3 | 1/3 |  | 1 |  | 0.2973 |
| SCN2B | Pre and post synaptic |  |  |  |  |  |  |  |  |  |  | 0.3355 |
| SCN3A | Presynaptic | 1 |  | 1 | 1 |  |  |  |  |  |  | 0.0010 |
| SCN3B | Presynaptic |  |  |  |  |  |  |  |  |  |  | NA |
| SCN4A |  |  |  | 1 | 1 |  |  |  |  |  |  | 0.0102 |
| SCN4B | Presynaptic |  |  |  |  |  |  |  |  | 1 |  | 0.4526 |
| SCN5A | Presynaptic | 2 |  |  | 2 |  |  |  | 1/0 |  |  | 0.5920 |
| SCN7A |  |  |  |  |  |  |  |  |  | 1 |  | 0.4564 |
| SCN8A | Presynaptic |  |  |  |  |  |  | 1/0 |  |  |  | 0.1961 |
| SCN9A | Presynaptic | 2 |  |  | 1 |  |  |  |  |  |  | 0.3597 |

**Table S9.** All genes in the voltage-gated sodium channel pathway and their hits/P-values in different genetic datasets. NS = nonsynonymous, LOF = loss-of-function, BG = Bulgarian, TW = Taiwanese, SZ = schizophrenia, T = transmissions, NT = non-transmissions, SynSysNet = Synaptic Proteins Database (<http://bioinformatics.charite.de/synsysnet/>)

1. **Enrichment of *de novo* mutations in voltage-gated sodium channels**

*De novo* mutations identified in exome sequencing studies of schizophrenia, autism spectrum disorder (ASD), intellectual disability (ID) and controls (ASD non-affected siblings and controls) were tested for enrichment among voltage-gated sodium channel genes using methods described previously^1^. Significant enrichments were found in both ID and ASD but not in schizophrenia and controls (Table S10). Significant associations were largely driven by *de novo* mutations in *SCN2A*. A breakdown of which voltage-gated sodium channel genes had *de novo* mutations is provided in Section 9 of the Supplementary Material, Table S9.

| **Phenotype** | Nonsynonymous | | | Loss-of-Function | | |
| --- | --- | --- | --- | --- | --- | --- |
|  | *P*_simulated_ | N mut. | Fold  enrichment | *P*_simulated_ | N mut. | Fold  enrichment |
| Schizophrenia | 0.68 | 1 | 0.87 | 0.14 | 1 | 6.35 |
| ASD | 0.015 | 5 | 3.5 | 0.0023 | 3 | 12.3 |
| ID | 5 × 10^-6^ | 5 | 19 | 3.6 × 10^-5^ | 3 | 47.8 |
| ASD sibs + controls | 0.48 | 1 | 1.5 | 1 | 0 | 0 |

**Table S10.** Statistical significance for enrichment of *de novo* mutations in voltage-gated sodium channel genes.

1. **Enrichments among voltage-gated sodium channel-interacting genes**

Compound heterozygosity in VGSC-interacting genes

Given strong genetic evidence implicating synaptic proteins in schizophrenia^1-3^, we postulated that the subset of synaptic proteins which interact with VGSCs would be enriched for additional classes of rare mutations. We therefore identified all VGSC-interacting proteins in the synaptic interactome available from the SynSysNet database (<http://bioinformatics.charite.de/synsysnet>). 10 of the 14 VGSCs were present in the SynSysNet database (Table S9). After excluding VGSCs that interacted with a different VGSC, 33 genes were found to interact with VGSCs (Table S11).

VGSC-interacting proteins were enriched for compound heterozygous genotypes (MAF ≤1%) among Bulgarian probands (*P*_permuted_ = 0.018, OR = 2, Supplementary Table S11). To test whether this enrichment was driven by a general synaptic excess of compound heterozygous genotypes in probands, we performed logistic regression which modelled proband/parent status against N compound heterozygous genotypes in VGSC-interacting proteins and included N compound heterozygous genotypes in the remaining 985 synaptic genes (genes from SynSysNet after excluding VGSCs and their interactions) and sequencing site/batch as covariates. Despite there being a non-significant excess of synaptic compound heterozygous genotypes in probands (MAF ≤1%, proband rate = 0.16, parent rate = 0.14, OR=1.16, 1-sided *P* = 0.16), the excess proband compound heterozygosity in VGSC-interacting genes remained significant after correcting for the general synaptic excess (*P_permuted_* = 0.032).

All VGSC-interacting genes disrupted by compound heterozygous genotypes in probands and parents interacted with a VGSC which itself was disrupted by a compound heterozygous genotype (i.e. our result showing proband enrichment of compound heterozygosity in VGSC-interacting genes would not change if we only included interactions with VGSCs that themselves were disrupted by compound heterozygous genotypes). The significance of this observation was evaluated by reassigning the 15 compound heterozygous genotypes in VGSC-interacting genes observed in probands (Supplementary Table S11) among all 33 VGSC-interacting genes, where the probability of a mutation occurring in a gene was proportional to its coding sequence length, and testing how many times all mutations fall within genes interacting with a disrupted VGSC. For 10,000 permutations, all 15 mutations occurred in genes interacting with a disrupted VGSC 634 times (*P* = 0.063).

A breakdown of the number of nonsynonymous compound heterozygous genotypes and *de novo* mutations in the Bulgarian trios, as well as gene-wide *P* values from a SCAT-O analysis of 5,585 independent schizophrenia cases and 8,103 independent controls, in synaptic proteins known to interact with VGSCs is provided in Table S11.

| **Gene** | **VGSC Interaction** | **Compound hets**  **≤1% frequency** | | **De novo mutation**  **(Missense/LOF counts)** | | | | **Exome chip**  ***P*** |
| --- | --- | --- | --- | --- | --- | --- | --- | --- |
|  |  | **Probands**  **N Mut.** | **Parents**  **N Mut.** | **SZ** | **ASD** | **ID** | **ASD sibling +**  **controls** |  |
| ANK3 | SCN1B, SCN2A,  **SCN5A** | 3 | 4 |  | 1/0 |  |  | 0.46 |
| AP1M1 | **SCN5A** |  |  |  |  |  |  |  |
| APOE | **SCN5A** |  |  |  |  |  |  |  |
| ATM | **SCN5A** | 1 | 1 |  |  |  |  | 0.33 |
| CALM3 | **SCN5A**, **SCN9A** |  |  |  |  |  |  |  |
| CNTN1 | SCN1B |  |  |  |  |  |  | 0.29 |
| CST3 | **SCN5A** |  |  |  |  |  |  |  |
| DLG1 | **SCN5A** |  |  | 1/0 |  |  |  | 0.35 |
| DLG2 | **SCN5A** |  |  | 0/1 |  |  |  | 0.25 |
| DLG3 | SCN2A, **SCN5A** |  |  |  |  |  |  |  |
| DLG4 | **SCN5A** |  |  |  |  | 1/0 |  | 0.45 |
| EEF1A1 | SCN1A |  |  |  |  |  |  |  |
| EEF1A2 | SCN1A |  |  |  |  | 1/0 |  |  |
| EEF2 | SCN1A |  |  |  |  |  |  |  |
| EXOC6 | **SCN5A** | 2 | 1 |  |  |  |  | 0.67 |
| GABBR1 | **SCN5A** |  |  |  |  |  |  |  |
| GNAO1 | SCN8A |  |  | 1/0 |  |  |  | 0.73 |
| KCNMA1 | **SCN5A** |  |  |  | 1/0 |  |  | 0.15 |
| LRP1 | **SCN3A** | 2 |  | 0/1 | 1/0 | 1/0 | 1/0 | 0.33 |
| MPP3 | SCN2A |  |  |  |  |  |  | 0.66 |
| NEDD8 | SCN1A |  |  |  |  |  |  |  |
| NFASC | SCN1B,SCN3B |  |  | 1/0 |  |  |  | 0.63 |
| PCDH1 | **SCN5A** |  |  |  |  |  |  | 0.26 |
| PHB | SCN8A |  |  |  |  |  |  |  |
| RPL13 | SCN1A |  |  |  |  |  |  | 0.12 |
| RPL29 | SCN1A |  |  |  |  |  |  |  |
| RPL6 | SCN1A |  |  |  |  |  |  |  |
| RPS16 | SCN1A |  |  |  |  |  |  |  |
| RYR2 | **SCN5A** | 1 | 1 |  |  |  |  | 0.81 |
| SACS | **SCN5A** | 4 | 6 |  |  |  |  | 0.03 |
| TLN2 | **SCN5A** | 2 | 2 |  |  |  |  | 0.12 |
| YWHAG | SCN1A |  |  |  |  |  |  |  |
| YWHAH | SCN1A |  |  |  |  |  |  |  |
| **Total**  **(Rate)** |  | **15**  **(0.025)** | **15**  **(0.012)** |  |  |  |  |  |

**Table S11**. Mutations in synaptic voltage-gated sodium channel-interacting genes. Column one displays synaptic genes which interact with a VGSC. Column two displays the VGSC that interacts with the gene displayed in column one. VGSCs in column two highlighted in bold are themselves disrupted by a proband compound heterozygous genotype. Column three and four show the number of compound heterozygous mutations in probands and parents, respectively. Columns five to eight show the number of *de novo* mutations observed in published reports of schizophrenia, ASD, ID and controls (ASD sibs + controls), respectively. Column nine shows gene-wide *P* values from our analysis of exome chip data.

Transmission disequilibrium tests in synaptic VGSC-interacting genes

The results from transmission disequilibrium tests for variants in synaptic proteins interacting with VGSCs are shown in Table S12. A significant deviation from the null was not observed for nonsynonymous variants with a MAF ≤1%, indicating a low false positive rate of variants called within these genes and that the excess compound heterozygosity in VGSC-interacting proteins is not driven by general over-transmission.

| **Variant (MAF)** | **Transmissions** | **Non-transmissions** | **P_uncorrected_** |
| --- | --- | --- | --- |
| NS (1%) | 512 | 492 | 0.53 |
| NS (Singletons) | 154 | 162 | 0.65 |
| LOF (1%) | 3 | 5 | 0.48 |
| LOF (Singletons) | 3 | 5 | 0.48 |

**Table S12.** Transmission disequilibrium test (TDT) of nonsynonymous (NS) and loss-of-function (LOF) variants in voltage-gated sodium channel-interacting genes.

*De novo* mutations in VGSC-interacting genes

We tested VGSC-interacting genes for enrichment of *de novo* mutations using previously described methods^1^. LOF *de novo* mutations were significantly overrepresented in VGSC-interacting genes in schizophrenia (Table S13). NS *de novo* mutations were also significantly overrepresented in ID and there was a trend for an excess of NS *de novo* mutations in schizophrenia (Table S13).

| **Phenotype** | **Nonsynonymous** | | | **Loss-of-function** | | |
| --- | --- | --- | --- | --- | --- | --- |
|  | ***P*_simulated_** | **N mut.** | **Fold**  **enrichment** | ***P*_simulated_** | **N mut.** | **Fold**  **enrichment** |
| Schizophrenia | 0.089 | 5 | 2.16 | 0.041 | 2 | 6.28 |
| ASD | 0.56 | 3 | 1.03 | 1 | 0 | 0 |
| ID | 0.016 | 3 | 5.74 | 1 | 0 | 0 |
| ASD sibs + controls | 0.76 | 1 | 0.71 | 1 | 0 | 0 |

**Table S13.** Statistical significance for enrichment of *de novo* mutations in voltage-gated sodium channel-interacting genes.

Exome chip analysis of synaptic VGSC-interacting genes

We did not find support associating synaptic VGSC-interacting genes with schizophrenia when analysed as a gene set in our exome chip data (*P* = 0.3).

1. **Phenotypic description of probands with a voltage-gated sodium channel compound heterozygous genotype.**

A small proportion (~14%) of the Bulgarian probands received a diagnosis of schizoaffective disorder. We tested whether the probands carrying a compound heterozygous genotype in a VGSC were overrepresented for having a diagnosis of schizophrenia or schizoaffective disorder. We also compared whether the mean age of onset and gender differed between probands carrying a VGSC compound heterozygous mutation and those that did not. None of these tests significantly deviated from the null (Table S14).

| **Test** | **Probands with a VGSC compHet** | **Probands without a VGSC compHet** | **2-sided *P* (test)** |
| --- | --- | --- | --- |
| Age of onset (Mean) | 20.13 | 23.85 | 0.12 (T test) |
| Diagnosis (Schizoaffective/schizophrenia) | 2/6 | 85/511 | 0.32 (Fisher’s Exact) |
| Gender (male/female) | 4/4 | 292/304 | 1 (Fisher’s Exact) |

**Table S14.** Phenotypic comparisons between Bulgarian probands with and without a voltage-gated sodium channel gene compound heterozygous genotype (MAF≤1%).

**References**

1. Fromer M, Pocklington AJ, Kavanagh DH, Williams HJ, Dwyer S, Gormley P *et al.* De novo mutations in schizophrenia implicate synaptic networks. *Nature* 2014; **506:** 179-184.

2. Purcell SM, Moran JL, Fromer M, Ruderfer D, Solovieff N, Roussos P *et al.* A polygenic burden of rare disruptive mutations in schizophrenia. *Nature* 2014; **506:** 185-190.

3. Kirov G, Pocklington AJ, Holmans P, Ivanov D, Ikeda M, Ruderfer D *et al.* De novo CNV analysis implicates specific abnormalities of postsynaptic signalling complexes in the pathogenesis of schizophrenia. *Mol Psychiatry* 2012; **17**(2)**:** 142-153.

4. Kumar P, Henikoff S, Ng PC. Predicting the effects of coding non-synonymous variants on protein function using the SIFT algorithm. *Nat Protocols* 2009; **4**(8)**:** 1073-1081.

5. Adzhubei IA, Schmidt S, Peshkin L, Ramensky VE, Gerasimova A, Bork P *et al.* A method and server for predicting damaging missense mutations. *Nat Meth* 2010; **7**(4)**:** 248-249.

6. Schwarz JM, Cooper DN, Schuelke M, Seelow D. MutationTaster2: mutation prediction for the deep-sequencing age. *Nat Meth* 2014; **11**(4)**:** 361-362.
